# Genome Sequence of Lactobacillus futsaii Y97, a Potential Probiotic Strain Isolated from Futsai of Taiwan

**DOI:** 10.1128/MRA.00747-19

**Published:** 2019-09-26

**Authors:** Xiaoyu Xu, Fang Yao, Qiufeng Gou, Fuying Dai, Qu Pan

**Affiliations:** a Department of Pathogenic Biology, Chengdu Medical College, Sichuan, China; Loyola University Chicago

## Abstract

Here, we report the complete genome sequence of Lactobacillus futsaii Y97, a potential probiotic strain isolated from futsai of Taiwan. The genome consists of one chromosome of 2.56 Mb and three plasmids. The genome contains 2,622 genes, which make up 87.06% of the genome.

## ANNOUNCEMENT

Lactobacillus futsaii is a Gram-positive rod-shaped nonmotile bacterium belonging to the lactic acid bacteria (1). Traditional fermented foods are rich sources of microorganisms that show probiotic and, hence, health-promoting characteristics (2, 3). *L. futsaii* shows the characteristics of acid resistance, bile salt tolerance, and intestinal colonization. Furthermore, it could be a new strain of probiotic starter for producing gamma-aminobutyric acid (GABA) (4).

*L. futsaii* Y97 was isolated from futsai, a traditionally home-brewed mustard product produced by Hakka tribes in Taiwan. The genome of *L. futsaii* Y97 was sequenced to identify its specific genetic components and explore its biological characteristics. *L. futsaii* Y97 was grown at 37°C for 48 h under static anaerobic conditions in MRS medium, which was usually used to isolate lactic acid bacteria. The culture was sent to the Beijing Genomics Institute (BGI; Shenzhen, China), and the genomic DNA was extracted using a MiniBEST bacterial genomic DNA extraction kit (TaKaRa, Dalian, China). The 2100 bioanalyzer (Agilent Technologies, Palo Alto, CA) was used to detect the quality of the genomic DNA of *L. futsaii* Y97, and then the genomic DNA was broken to pieces with a desired size by a Covaris S/E210 or g-TUBE instrument. A 270-bp insert library with a read length of 2 × 150 bp was constructed using a PCR protocol, after the blunt phosphorylated adapters were ligated to the ends of the DNA fragments, and sequenced using the Illumina HiSeq 4000 platform. A 10-kb template library was constructed using a SMRTbell protocol, by which both ends of the DNA fragment were ligated to the connector of the hairpin structure. The 10-kb template library was sequenced using the PacBio Sequel platform. Four single-molecule real-time (SMRT) cell zero-mode waveguide arrays of sequencing were used to generate the subread set. After filtering subreads (length, <1 kb) and adapter sequences, SMRT sequencing generated 4,878,353,482 bp of clean data, with a total of 494,569 subreads and a mean read length of 9,863 bp. The subreads were used for *de novo* assembly with Hierarchical Genome Assembly Process (HGAP) version 3 in SMRT analysis version 2.3.0 software (https://www.pacb.com/documentation/smrt-analysis-software-installation-v2-3-0/) (5), which yielded four contigs with an *N*_50_ value of 11,995 bp. Correction of the PacBio assembly was performed by soapSNP and soapIndel software with default parameters using 851 Mb of clean data from the Illumina HiSeq 4000 sequencing, which were obtained by removal of low-quality reads and adapters and duplication contamination from 1,246 Mb of raw reads (6). The single-base quality of the genome reached 0.9999 after being polished with Quiver. The genome coverage values were 321× with the Illumina HiSeq 4000 platform and 1,841× with the PacBio Sequel platform. Gene prediction was performed with Glimmer version 3.02 (http://ccb.jhu.edu/software/glimmer/index.shtml) (7). The functional annotation was accomplished by BLAST with the nonredundant (NR) database, Swiss-Prot (https://www.uniprot.org/uniprot/?query=reviewed:no%20taxonomy:1423818), Trembl (https://www.uniprot.org/uniprot/?query=reviewed:no%20taxonomy:1423818), Antibiotic Resistance Genes Database (ARDB) (https://card.mcmaster.ca/), Pathogen Host Interactions (PHI) (http://www.phi-base.org/), COG (https://www.ncbi.nlm.nih.gov/COG/), Carbohydrate-Active enZYmes (CAZy) database (http://www.cazy.org/), KEGG database (http://www.genome.jp/kegg/), and Gene Ontology (GO) database (http://geneontology.org/) (8–14).

The complete genome of *L. futsaii* Y97 consists of a circular chromosome of 2,558,218 bp and three circular plasmids (37,880 bp, 27,087 bp, and 25,584 bp), with G+C contents of 35.68%, 39.44%, 38.93%, and 34.80%, respectively. The genome contains 2,622 genes; the total length of the genes is 2,306,070 bp, which makes up 87.06% of the genome. The number of tandem repeat sequences is 71; the total length of the tandem repeat sequences is 4,575 bp, which makes up 0.1727% of the genome. Also, 43 minisatellite DNAs, 3 microsatellite DNAs, 56 tRNAs, 12 rRNAs, and 7 small RNAs (sRNAs) were predicted. The genome sequence information presented here will help further specific studies of this strain and to exploit its probiotic potential.

### Data availability.

The genome sequence was deposited in GenBank (BioProject accession number PRJNA545382) under accession numbers CP040736, CP040737, CP040738, and CP040739 and SRA accession numbers SRR9164841 and SRR9157803. The versions described in this paper are the first versions.
